# Th1, Th17, and Treg Responses are Differently Modulated by TNF-α Inhibitors and Methotrexate in Psoriasis Patients

**DOI:** 10.1038/s41598-019-43899-9

**Published:** 2019-05-17

**Authors:** Sandro C. Furiati, Jonatas S. Catarino, Marcos V. Silva, Rafaela F. Silva, Rayane B. Estevam, Reginaldo B. Teodoro, Sanivia L. Pereira, Meire Ataide, Virmondes Rodrigues, Denise B. R. Rodrigues

**Affiliations:** 1 University of Uberaba, Uberaba, Minas Gerais Brazil; 2 Laboratory of Immunology, Department of Biological Sciences, and Cefores, Triângulo Mineiro Federal University, Uberaba, Minas Gerais Brazil

**Keywords:** Inflammatory diseases, Chronic inflammation

## Abstract

Psoriasis is a chronic, recurrent, immune-mediated, hyperproliferative inflammatory skin disease. The role of the adaptive immune system, particularly of Th1 and Th17 lymphocytes, has been regarded as prominent in the immunopathogenesis of psoriasis, as well as decreased Tregs function. Immunobiological drugs were administered in therapeutic pulses and a few studies evaluate their effects on the immune repertoire. The aim of this study was to evaluate the adaptive immune profile of patients with severe psoriasis under immunobiological treatment in two time points. Thirty-two psoriasis patients and 10 control patients were evaluated. In the group of psoriasis patients, 10 patients were on anti-TNF and 14 patients on methotrexate treatment, while 8 individuals were not treated. IL-17, IFN-γ, TNF-α, IL-6, IL-2, and IL-10 were analyzed. CD4 T cell intracellular cytokines were analyzed. It was observed that stimulation could significantly increase the production of IL-17, IFN-γ, TNF-α, and IL-10 only before anti-TNF pulse therapy. The activation of Th1 and Treg cells after stimulation was significantly higher before anti-TNF pulse. Patients on methotrexate or anti-TNF therapy produced significantly lower levels of TNF-α, IL-10, and IL-6. Furthermore, these patients showed a significant decrease in the activated CD4+ T cells. The treatment with immunomodulator or methotrexate modulates the activation of CD4+ T cells, and anti-TNF treatment appears to have a modulating effect on the activation and production of Th1, Th17, and Treg cells.

## Introduction

Psoriasis is a chronic inflammatory skin disease that affects about 2–3% of the world’s population^1–3^. It is caused by a combination of genetic and environmental factors, as well as by complex interactions between the innate and adaptive immune system^4^ There are several types of psoriasis, and the clinical variant called psoriasis vulgaris or plaque psoriasis is the most common, affecting 85–90% of the patients^5^. Up to one third of these patients may be associated with the articular manifestation of the disease, called arthropathic psoriasis^6^. Clinically, psoriasis vulgaris is usually characterized by lesions on well-demarcated round to oval erythematous plaques, with thick, dry, silvery adherent scales^7^. These scales are the result of a hyperproliferative epidermis in which keratinocytes mature early, leading to an incomplete cornification with the retention of keratinocyte nuclei in the stratum corneum (parakeratosis). This results in epidermal hyperplasia (acanthosis) and elongation of papillary ridges^8^. Histologically, these lesions appear as an inflammatory infiltrate, consisting mainly of dendritic cells (DCs), macrophages, and T cells in the dermis, as well as neutrophils and some T cells in the epidermis. The erythematous characteristic of the lesions is due to the increase in the number of tortuous capillaries that reach the skin surface through papillary ridges^9^.

IFN-α and other cytokines produced by innate immune system cells, such as interleukin (IL)-1, IL-6, tumor necrosis factor alpha (TNF-α), and IFN-γ, stimulate the activation and maturation of myeloid DCs, which are considered the key cells that link the innate immune system and the adaptive immune system^10^. In addition to being activated by these cytokines, myeloid DCs also have TLRs (Toll-like receptors), through which they can directly interact with the auto-RNA–LL-37 complex, causing their concomitant activation, thus starting to produce several proinflammatory cytokines (IL-23, TNF-α, IL-12, IL-6, IL-20, and IL-1)^11^.

The mature myeloid DCs migrate to the lymph nodes, where, with the help of cytokines they produce, they induce the differentiation of naïve CD4^+^ T cells (Th0) into Th17, Th1, or Th22, thus triggering the activity of the adaptive immune system in the inflammatory process of psoriasis^12^. These differentiated CD4^+^ T cells migrate to the dermis, where, by interacting with the innate immune system cells, they produce their typical inflammatory cytokines^13^. Th17 cells produce IL-17, IL-6, TNF-α, IL-21, and IL-22^14^; Th1 cells produce TNF-α and IFN-γ^15^; and Th22 cells produce IL-22 and TNF-α^16^. The inflammatory process is initially dominated by Th17 cells, but in stable psoriasis plaques, it subsequently shifts towards a process dominated by IFN-γ-producing Th1 cells^17,18^.

Keratinocytes are the main targets of these cytokines, especially of IL-17, IFN-γ, TNF-α, and IL-22, which directly or indirectly lead to their hyperproliferation, as well as induce them to produce various other cytokines, chemokines, and antimicrobial peptide, which in turn continue to stimulate the activation and recruitment of cells of the innate and adaptive immune system to the lesions, thus perpetuating the inflammatory process of psoriasis^9^. IL-17 and IL-23 are considered the key cytokines in the immunopathogenesis of psoriasis, and they are referred to as the IL-23–IL-17 axis^19^. In the immunopathogenesis of psoriasis, TNF-α promotes the activation and maturation of DCs, which, in turn, produce more IL-23^20^.

Regulatory T cells (Tregs), identified as CD4^+^ CD25^+^ Foxp3^+^ cells, play a crucial role in immune tissue homeostasis and in self-tolerance because they are capable of suppressing effector cell activation and proliferation through the production of IL-10, hence controlling inflammatory processes in the body^21^. However, in psoriasis, this regulatory capacity is diminished or absent^22^. Studies indicate a malfunction of Tregs or a decrease in their numbers in psoriatic lesions^23^. T regulatory cells expressing mLAP (membrane latency-associated peptide) is a population of human Tregs that is different from classic CD4+Foxp3+CD25 high natural Tregs^24^. LAP is a propeptide that favors the release of TGF-β into the extracellular milieu and for this reason is related to several functions in T regulatory cells such as suppressive activity, survival and peripheral conversion of iTregs and Th17^25^. T regulatory cells expressing mLAP shows a potent immunosuppressive activity and are augmented in some neoplasic disorders, such as lung adenocarcinoma^26^ and Colorectal Cancer^27^.

Systemic treatment of psoriasis is reserved for moderate and severe cases, and it consists in the use of medications that can inhibit or reduce the immune response, thus reducing inflammation and inflammatory cell infiltration, as well as improving psoriatic lesions^28^. Methotrexate and anti-TNF are the major immunosuppressive medications used in the systemic treatment of psoriasis^29^. Methotrexate is a folic acid analogue and antimetabolite that has an effect on both circulating and cutaneous lymphocytes^30^. *In vitro*, keratinocytes are more resistant to its cytotoxic effects than T cells, thus proving its immunosuppressive properties^31^. It is considered to be antineoplastic, antipsoriatic, and antirheumatic^32^, and it inhibits neutrophil chemotaxis and the release of TNF-α, IFN-γ, IL-12, and IL-6, ultimately leading to its anti-inflammatory activity^33^. Anti-TNF is used for treating psoriasis in case of therapeutic failure or contraindication of methotrexate. Immunobiologicals (IBs) can be used alone or, in some cases, concomitantly with methotrexate, and there are five immunobiologicals currently approved for the treatment of psoriasis in Brazil, three of which are anti-TNF drugs (adalimumab, infliximab, and etanercept) and two interleukin inhibitors (ustekinumab and secukinumab). Ustekinumab is also an inhibitor of IL-23, and secukinumab is also an inhibitor of IL-17A^34^.

Anti-TNF agents block the soluble fraction of TNF-α and the transmembrane fraction of TNF that is expressed on the membrane of cells producing this cytokine^35^. TNF-α blockade decreases the activation of myeloid DCs, which are important sources of IL-23 and play a vital role in the differentiation of naïve CD4^+^ T cells into Th17 cells^28^. After TNF-α blockade, the activation of these cells is also decreased, and so is the differentiation of Th0 into Th1 or Th22 and the production of their respective standard cytokines^36^. A study of 21 patients using anti-TNF adalimumab in China showed a decrease in the circulating populations of Th17, Th1, and Th22 cells^37^. In addition to these effects, the number of CD4^+^ CD25^+^ Foxp3^+^ cells increased in the peripheral blood of psoriasis patients after treatment with biologicals. This increase was associated with a good clinical response and a reduction in the psoriasis area severity index (PASI), although some patients had decreased numbers of Tregs after the biological treatment, which was associated with worsening of the disease.

However, the effect of TNF-blocker therapy at different intervals of the therapeutic cycle has not yet been clarified. This knowledge is necessary to understand the reasons for the low therapeutic response and the short duration of its effects in some individuals. Therefore, the aim of the present study was to evaluate the pattern of the adaptive immune response in patients with stable severe psoriasis without treatment and patients undergoing systemic therapy with methotrexate or anti-TNF agents at different intervals of the therapeutic cycle.

## Materials and Methods

### Patients

Thirty-two patients with stable severe psoriasis undergoing treatment at the dermatology clinic, who agreed to participate in this study, were recruited. The diagnosis was confirmed by biopsy. Ten patients had previous assessment of the PASI, with a result above 10, and were regularly using anti-TNF immunobiological medication (adalimumab); 14 patients had previous diagnosis of severe psoriasis (PASI > 10) and had been on methotrexate treatment for at least 2 months; 8 patients had previous diagnosis of severe psoriasis (PASI > 10) and had not been on therapy for at least 2 months. Patients’ mean age and gender distribution are presented at Table 1. Ten healthy control subjects were selected paired by gender. The study was approved by the Ethics Committee (*CAAE*) of UNIUBE, under the protocol number 63049316.1.100005145, and was conducted at the University of Uberaba and at the Clinical Hospital (*Hospital das Clínicas*) of the Federal University of Triângulo Mineiro (UFTM), Uberaba, Minas Gerais, Brazil. The patients were subjected to blood collection for cell culture, with the aim of analyzing the production of cytokines in the culture supernatant and the T cell phenotype by flow cytometry. The patients who used IBs were submitted to two blood samplings; one immediately before receiving the therapeutic dose (day 0) and the other in the middle of the cycle (day 7), between applications. All the methods were performed in accordance with both institutional guidelines and all individuals who accepted to participate in this study signed an informed consent form after clarification.

**Table 1:** Gender and age mean of patients and healthy subjects.

	Gender (Male/Female)	Age – mean (Male/Female)
Healthy subjects	5/5	41.1/42.8
Patients without therapy	6/2	44.6/56.5
Patients treated with methotrexate	7/7	52.8/46.6
Patients treated with adalimumab	5/5	43.2/46.1

### Isolation and culture of peripheral blood mononuclear cells

Peripheral blood mononuclear cells (PBMCs) were isolated by density gradient centrifugation, using Histopaque-1077 (Sigma-Aldrich, St Louis, Mo, USA), for 30 mins at 400 × *g*, at 24 °C. The cells were then resuspended in RPMI 1640 medium (Sigma-Aldrich) containing 50 mM HEPES buffer (Gibco, Grand Island, NY, USA), 10% of inactivated fetal bovine serum (Gibco), 2 mM of L-glutamine (Gibco), and 40 mg/mL of gentamicin (Neoquímica, Anápolis, Brazil) to a final concentration of 2 × 10^6^ cells/mL. PBMCs were cultured in 24-well microplates (Falcon, San Jose, CA, USA) in the presence or absence of anti-CD3 and anti-CD28 for 48 h at 37 °C in a 5% CO_2_ atmosphere. Cells (for immunophenotyping) and supernatants (for cytokine measurement) were collected after 48 h. The cells were collected after 48 h for immunophenotyping, and the supernatants were collected after 48 h and stored at −70 °C for the measurement of soluble mediators using the cytometric bead array (CBA) (BD Bioscience, San Diego, CA, USA).

### Flow cytometry for analysis of the expression of surface molecules, cytokines, and transcription factors

For the analysis of the expression of surface molecules, cytokines, and transcription factors in cells derived from the 48 h culture, cells were washed for 10 min at 400 g, at 4C, in Hanks’ medium supplemented with 10% human AB^+^ serum, previously submitted to inactivation of the complement system, and incubated for 30 mins. The cells were split in three tubes and then labeled with antibodies targeting surface molecules: tube 1 - anti-CD4–PE Cy5 and anti-CD69–PE, tube 2 - anti-CD4–PE Cy5, anti-CD25–FITC, and anti-h/m-LAP–APC (BD Pharmingen) or tube 3 - respective control isotypes antibodies, and maintained at 4C for 30 mins. After that, the cells were washed three times for 10 min at 400 × *g* and 4 °C to remove excess antibodies, resuspended in 500 μL PBS containing 0.5% paraformaldehyde, and stored at 4C in a dark chamber until flow cytometry analysis. For intracellular detection, the cells were fixed and permeabilized with 250 μL of Cytofix/Cytoperm (BD Biosciences) at 4C for 30 mins. Next, they were washed three times in Perm/Wash (BD Biosciences), containing 10% fetal bovine serum (Sigma-Aldrich). In tube 1 were added anti-FoxP3–PE, in tube 2 anti-IL-17–Alexa Fluor 488, and anti-IFN-γ–Alexa Fluor 647 and in tube 3 respective intracellular isotype control antibodies. The cells were incubated at 4C for 30 min. At the end of this period, the cells were washed in Perm/Wash three more times for 10 mins at 400 g, at 4 °C, resuspended in 200 μL of 0.5% paraformaldehyde and stored in a dark chamber at 4C until flow cytometry analysis. Two tubes were placed parallel to each labeled sample: A tube without antibodies and a tube containing control isotopes compatible with the fluorescence used. Data acquisition (50,000 events/tube) was performed using a FACSCalibur cytometer (BD Biosciences), using the CellQuest software (BD Biosciences). Data analysis was performed using FlowJo 10.0.6 software (Tree Star) by isolating leukocyte populations through gates established according to the size (FSC) and granularity (SSC) characteristics of T cell populations.

### Cytokine concentrations in the culture supernatants

Production of IL-17A, IFN-γ, TNF-α, IL-10, IL-6, and IL-2 was analyzed simultaneously in the culture supernatants of PBMCs, using the CBA Human Inflammatory Cytokine Kit (BD Biosciences), according to the manufacturer’s instructions. The samples and recombinant cytokines were incubated with microspheres of different fluorescence intensities conjugated with captured antibodies specific for each cytokine. Then, PE-conjugated antibodies specific for each cytokine were added. After incubation, the microspheres were washed with the corresponding solutions and analyzed on a FACSCalibur cytometer (BD Biosciences) using the CellQuest software (BD Biosciences). The microspheres specific for each cytokine were separated due to the fact that they emitted different intensities of fluorescence at 660 nm, and the amount of cytokines conjugated with each of them was separated by fluorescence intensity at 585 nm. Sample data and data on recombinant cytokines were collected and subsequently analyzed using FCAP Array 2.0 software (Soft Flow, Pécs, Hungary), and cytokine concentrations were determined using standard curves.

### Statistical analysis

Statistical analysis was performed using the GraphPad Prism software (version 6.00; GraphPad Software, La Jolla, CA, USA). The Wilcoxon Signed Rank Test was used to compare two continuous variables in the same patients. The Kruskal-Wallis test was used to compare three or more groups, followed by Dunn’s post-hoc test. The difference was considered significant when p < 0.05.

## Results

### Treatment with anti-TNF downregulates the production of IL-17A, IFN-γ, TNF-α, and IL-10

**Figure 1:**
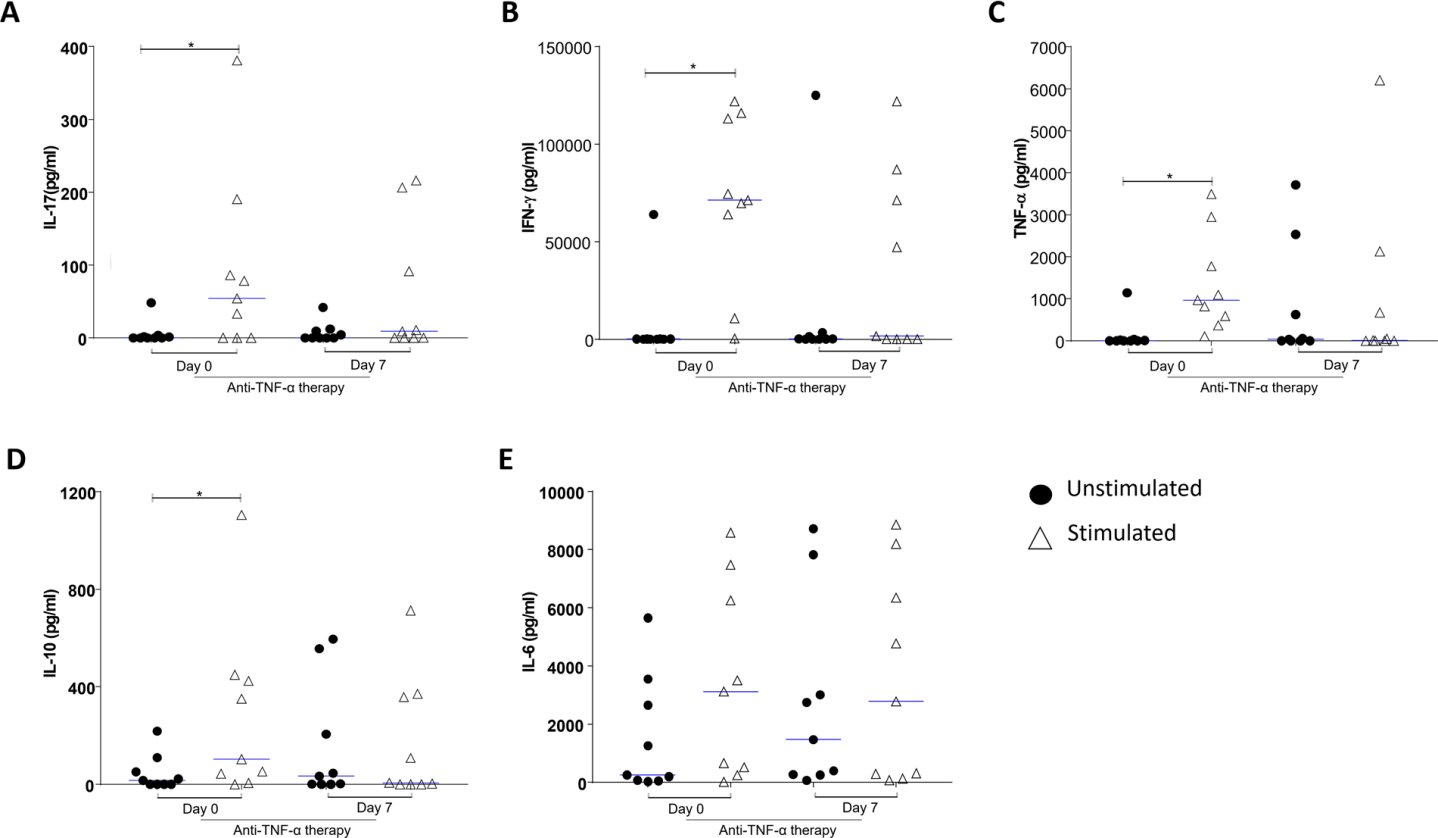
Analysis of cytokines in culture supernatant of PBMC, incubated for 48 h in the presence of medium (unstimulated) or with anti-CD3 and anti-CD28 mAbs (stimulated). The PBMCs were obtained prior to pulse therapy (day 0) and during pulse therapy (day 7) of patients with severe plaque psoriasis treated with anti-TNF agents. (**A**) IL-17 levels (*Wilcoxon; p < 0.05), (**B**) IFN-γ levels (*Wilcoxon; p < 0.05), (**C**) TNF-α levels (Wilcoxon; p > 0.05), (**D**) IL-10 levels (*Wilcoxon; p < 0.05), (**E**) IL-6 levels (*Wilcoxon; p < 0.05). The results are expressed in pg/mL. *Indicates statistical significance.

Cytokine analyses of psoriatic patients on anti-TNF therapy were performed on two occasions: prior to pulse therapy (day 0) and 7 days after the anti-TNF therapy (day 7). IL-17, IFN-γ, TNF-α, IL-10, IL-6, and IL-2 levels were analyzed by CBA of the PBMC culture supernatant 48 h after stimulation with anti-CD3 and anti-CD28 or after no stimulation (Fig. 1). Analysis of IL-17 levels in the PBMC supernatants showed that anti-CD3 and anti-CD28 stimulation significantly increased IL-17 production just before the pulse therapy (day 0), compared to the untreated supernatants (p = 0.031) (Fig. 1A). On the other hand, there was no significant increase in IL-17 production after anti-CD3 and anti-CD28 stimulation of the supernatants of patients whose pulse therapy was in progress (day 7) (Fig. 1A). Similarly, anti-CD3 and anti-CD28 stimulation significantly increased IFN-γ production only before pulse therapy (day 0), when compared with the untreated supernatants (day 0) (p = 0.007) (Fig. 1B). There was no significant increase in IFN-γ production after anti-CD3 and anti-CD28 stimulation of the supernatants of patients whose pulse therapy was in progress (day 7) (Fig. 1B). Similar to IFN-γ, anti-CD3 and anti-CD28 stimulation could significantly increase TNF-α production only before pulse therapy (day 0), compared with the untreated supernatants (day 0) (p = 0.007) (Fig. 1C). However, when TNF-α levels were observed under anti-CD3 and anti-CD28 stimulation, there was no significant difference between the levels in the supernatants of patients whose pulse therapy was in progress (Fig. 1C). When evaluating IL-10 levels, anti-CD3 and anti-CD28 stimulation significantly increased IL-10 production in the supernatants of patients who had not undergone pulse therapy (day 0), when compared to the untreated supernatants (day 0) (p = 0.0078) (Fig. 1D). There was no significant increase in IL-10 production after anti-CD3 and anti-CD28 stimulation of the supernatants of patients whose pulse therapy was in progress (day 7) (Fig. 1D). No significant upregulation was observed in the levels of IL-6 (Fig. 1E).

### Treatment with anti-TNF downregulates T cell activation

**Figure 2:**
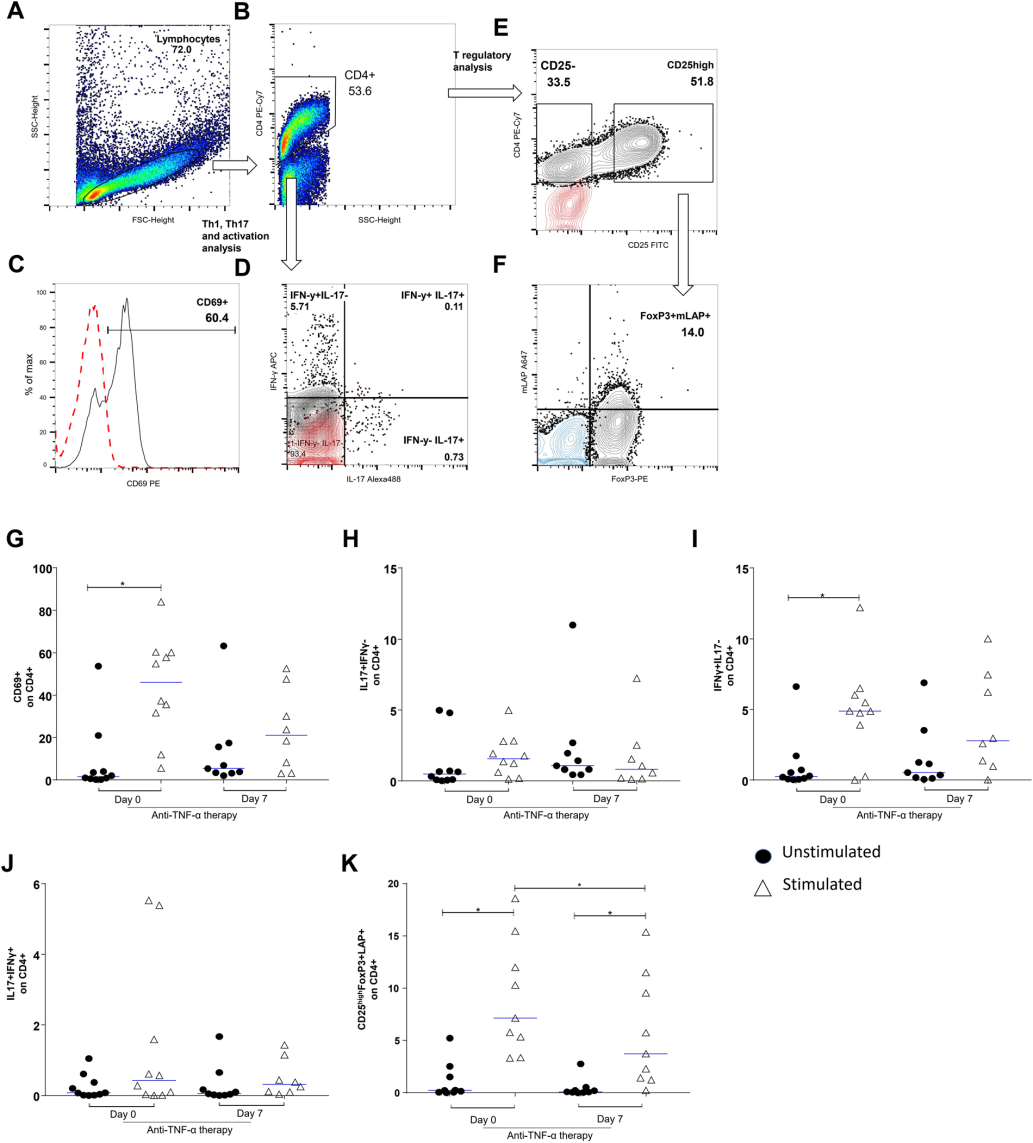
Flow cytometric analysis of cell activation markers and intra-cellular cytokine expression on CD4+ T-lymphocytes (TL): PBMCs were obtained prior to pulse therapy (day 0) and during pulse therapy (day 7) of patients with severe plaque psoriasis treated with anti-TNF agents. PBMC, cultured for 48 h in the presence of medium (unstimulated) or with anti-CD3 and anti-CD28 mAbs (stimulated). After recovered, PBMC were incubate with appropriate mAbs and isotype controls. Panels A to F show gates strategies for analysis. In panels C–E the red dots indicate the labeling of the control isotypes. In panel F, the blue dots indicate the CD25^−^ population. Panel G: CD69^+^ on CD4+ TL (Wilcoxon p < 0.05), panel H: IL-17^+^IFN-γ^−^ on CD4+ LT (Wilcoxon p > 0.05), panel I: IL-17^−^ IFN-γ^+^ on CD4+ LT (Wilcoxon p < 0.05), panel J: IL-17^+^IFN-γ^+^ on CD4+ LT (Wilcoxon p > 0.05) and panel K: CD25^high^FoxP3^+^LAP^+^ on CD4+ LT (Wilcoxon p < 0.05). The results are expressed in percentage on the CD4+ TL. *Indicate statistical significance.

The response of T cells after anti-CD3 and anti-CD28 stimulation and CD4 T cells were analyzed by the expression of the cell activation marker CD69 and intracellular cytokines. T cells obtained from PBMCs before pulse therapy (day 0) and 7 days after the anti-TNF therapy (day 7) were analyzed by flow cytometry after 48 h of culture. As for cell activation markers, the expression of CD69 in CD4^+^ T cells stimulated with anti-CD3 and anti-CD28 was significantly higher in patients before initiating pulse therapy (day 0) than in the same patients, but without cell stimulation (p = 0.02) (Fig. 2G). There was no significant difference between IL-17-producing CD4^+^ cells (IL-17^+^IFN-γ^−^) that were or were not stimulated with anti-CD3 and anti-CD28 (Fig. 2H). Nonetheless, when comparing IFN-γ-producing CD4^+^ cells (IL-17ˉIFN-γ^+^) stimulated with anti-CD3 and anti-CD28 before pulse therapy (day 0), there was a significant increase in the percentage of IFN-γ-producing cells compared with the non-stimulated cells (day 0) (p < 0.013) (Fig. 2I). On the other hand, there was no significant difference when comparing CD4^+^IFN-γ^+^ cells and IL-17-producing cells (IL-17^+^ IFN-γ^+^) with or without stimulation (Fig. 2J). CD4 T lymphocytes CD25^hi^FoxP3^+^ LAP^+^ showed a significant increase in the percentage of Treg cells after stimulation with anti-CD3 and anti-CD28 before (day 0) and after (day7) pulse therapy (day 0) compared with the percentage of cells in the same patients, but without stimulation (p = 0.003). Furthermore, before pulse therapy (day 0), this response was significantly higher than at after (day7) (p = 0.004) (Fig. 2K). Figure 2A–F illustrate the acquisition strategies. In Fig. 2C–E, the red dots indicate the labeling of the control isotypes. In Fig. 2F, the blue dots indicate the CD25^−^ population.

### Treatment with anti-TNF or methotrexate downregulates the production of cytokines in the culture supernatants

**Figure 3:**
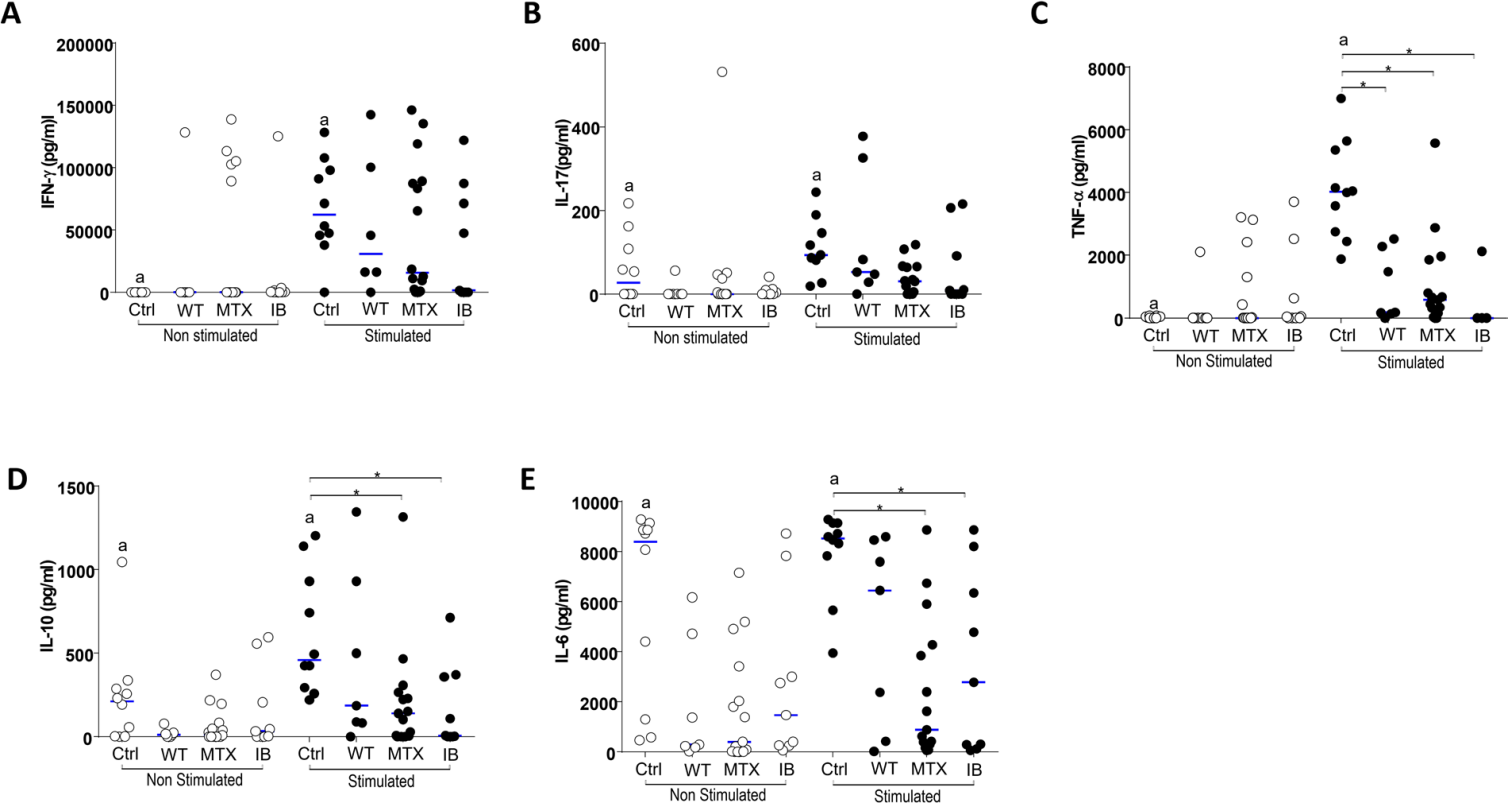
Analysis of cytokines in culture supernatant of PBMC, incubated for 48 h in the presence of medium (unstimulated) or with anti-CD3 and anti-CD28 mAbs (stimulated). The PBMCs were obtained from healthy subjects (Ctrl), psoriasis patients without therapy (WT), patients treated with methotrexate (MTX), and patients treated with immunobiologicals (IB). Panel A: IFN-γ levels (^a^Wilcoxon; p < 0.05), panel B: IL-17 levels (^a^Wilcoxon p < 0.05), panel C: TNF-α levels (^a^Wilcoxon; p < 0.05 and *Kruskal-Wallis p < 0,05), panel D: IL-10 levels (^a^Wilcoxon p < 0.05 and *Kruskal-Wallis p < 0,05), panel E: IL-6 levels (^a^Wilcoxon; p = 0.05 and *Kruskal-Wallis p < 0,05). The results are expressed in pg/mL. a and *indicate statistical significance.

Psoriasis patients were grouped as follows: patients without any therapy, patients treated with methotrexate, and patients treated with anti-TNF. The effects of anti-TNF therapy were analyzed 7 days after initiation of the therapy. There were no significant differences in IFN-γ and IL-17 levels in the supernatants between the different patient groups. However, only the control group showed significant upregulation of IFN-γ and IL-17 when stimulated, compared with the unstimulated cultures (Fig. 3A,B). TNF-α production after anti-CD3 and anti-CD28 stimulation was significantly lower in untreated psoriatic patients, in patients treated with methotrexate, and in patients treated with anti-TNF, when compared to healthy control individuals (p = 0.017, p = 0.004, and p = 0.003, respectively). Furthermore, when stimulated, only the control group presented significant upregulation of IFN-γ and IL-17 compared with the unstimulated cultures (Fig. 3C).

It was observed that, after anti-CD3 and anti-CD28 stimulation, IL-10 levels were significantly lower in psoriasis patients treated with methotrexate and in patients on anti-TNF treatment than in healthy control subjects (p = 0.029 and p = 0.030, respectively) (Fig. 3D). Again, only in the control group was the anti-CD3 and anti-CD28 stimulation able to induce a significant production of IL-10 (Fig. 3D).

Similarly, IL-6 levels were significantly lower in patients treated with methotrexate or treated with anti-TNF in comparison to healthy controls (p = 0.002 and p = 0.033, respectively). Here, patients without any therapy showed a significant upregulation of IL-6 after anti-CD3 and anti-CD28 stimulation (p = 0.001) (Fig. 3E).

### Treatment with anti-TNF and methotrexate modulates CD4^+^ T cell activation

**Figure 4:**
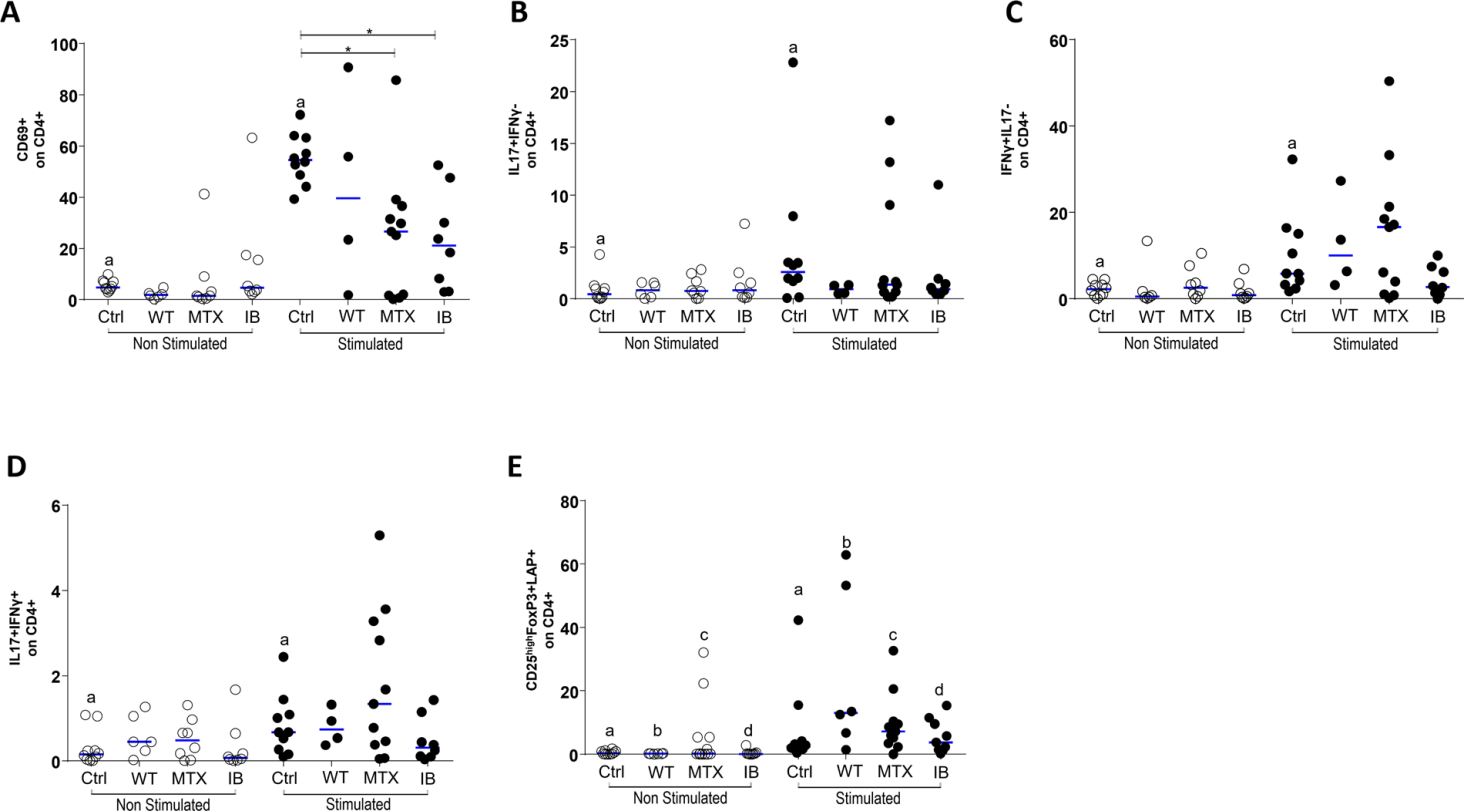
Flow cytometric analysis of cell activation markers and intra-cellular cytokine expression on CD4+ T-lymphocytes (TL): The PBMCs were obtained from healthy subjects (Ctrl), psoriasis patients without therapy (WT), patients treated with methotrexate (MTX), and patients treated with immunobiologicals (IB). PBMC, incubated for 48 h in the presence of medium (unstimulated) or with anti-CD3 and anti-CD28 mAbs (stimulated). After recovered, PBMC were incubate with appropriate mAbs and isotype controls. Panel A: CD69^+^ on CD4+ TL (^a^Wilcoxon; p = 0.05 and *Kruskal-Wallis p < 0,05), panel B: IL-17^+^IFN-γ^−^ on CD4+ LT (^a^Wilcoxon p > 0.05), panel C: IL-17^−^ IFN-γ^+^ on CD4 + LT (^a^Wilcoxon p < 0.05), panel D: IL-17^+^IFN-γ^+^ on CD4 + LT (^a^Wilcoxon p > 0.05) and panel E: CD25+ FoxP3^+^LAP^+^ on CD4+ LT (^a^Wilcoxon; p 0.05 and *Kruskal-Wallis p < 0,05). The results are expressed in percentage on the CD4+ TL. A and *indicates statistical significance.

In patients grouped according to treatment, the response of T cells after anti-CD3 and anti-CD28 stimulation was analyzed by the expression of the cell activation markers, CD69 and CD4, and intracellular cytokines. It was observed that the expression of CD69 in CD4^+^ T cells after anti-CD3 and anti-CD28 stimulation was significantly lower in patients on methotrexate and in patients receiving anti-TNF than in healthy control subjects (p = 0.009 and p = 0.02, respectively). Only in the control group was the anti-CD3 and anti-CD28 treatment able to significantly upregulate the number of activated CD4^+^ T cells (p = 0.002) (Fig. 4A).

There was no significant difference in the percentages of IL-17-producing helper T cells (IL-17^+^IFN-γ^−^), IFN-γ-producing cells (IL-17^−^IFN-γ^+^), or IFN-γ^+^IL-17^+^ double-positive CD4^+^ cells among the different groups of patients. However, in control subjects, anti-CD3 and anti-CD28 stimulation was able to significantly upregulate the number of IFN-γ^+^, IL-17^+^, and IFN-γ^+^IL-17^+^ double positive CD4^+^ T cells (p = 0.01, p = 0.01, and p = 0.01, respectively) (Fig. 4B–D). On the contrary, it was observed that, after stimulation with anti-CD3 and anti-CD28, there was a significant increase in CD4^+^ T cells of a regulatory phenotype (CD25^hi^FoxP3^+^LAP^+^) in untreated psoriasis patients compared with the group treated with methotrexate (p = 0.03) (Fig. 4F).

## Discussion

Psoriasis is an immune-mediated skin disease with various clinical subtypes of different pathogenesis; the patients enrolled in this study had stable plaque psoriasis. The major advances in the treatment of severe psoriasis were made with the introduction of biological products. However, only long-term results of clinical trials and post-marketing surveillance can demonstrate the success of these new therapies^38^. Understanding the mechanisms of the actions of these products is of great importance for choosing the appropriate therapy and so are the suspension criteria or changes in the course of the treatment. The complex interactions of innate and adaptive immune system cells, in conjunction with the molecules released during the immune response, may trigger the modulation of elements other than those that are targeted during a therapy. Thus, several cytokines have been studied as blocking targets in biological therapies.

IL-17A is considered the key cytokine in the inflammatory process of psoriasis because it has a direct influence on the activation and hyperproliferation of keratinocytes^39,40^. There is a decrease in the expression of this cytokine *in situ* when biological therapies are used to treat moderate to severe psoriasis and, although Th17 cell products are modulated rapidly during the course of treatment with biological therapy, Th1 and Th2 cell products are modulated at a later phase, months after the disease has significantly improved^28^. Similarly, IL-12 and IL-23 inhibitors prevent Th1 and Th17 cells to secret their cytokines, thus decreasing the production of IFN-γ and IL-17A, respectively^40^.

In this study, we analyzed 32 patients with psoriasis; ten of them under treatment with TNF inhibitors, receiving one dose each 15 days. All patients, on the day preceding the therapeutic pulse (day 0), presented more psoriasis symptoms than on day 7, after receiving TNF inhibitors. These clinical signs are important and indicative of the need to administer another dose and the effectiveness of the treatment.

In the present study, it was observed that the stimulation of cells from patients with anti-CD3 and anti-CD28 led to a heterogeneous response, which may be related to the genetic diversity of human population. Although heterogeneous, this response led to a significant increase in IL-17A levels only in day 0, on which the assays were performed just before anti-TNF pulse therapy. On the contrary, when stimulating these cells with anti-CD3 and anti-CD28, no significant increase in IL-17A levels 7 days after anti-TNF therapy was observed. Investigating the number of IL-17^+^CD4^+^ T lymphocytes, we observed that anti-CD3 and anti-CD28 stimulation did not induce a significant increase in the number of IL-17-producing cells in both samples, suggesting that the TNF blockade consistently inhibited the generation of Th17 cells.

Our results are in agreement with the literature, which shows that the use of anti-TNF and other cytokine inhibitors such as anti-IL-12/anti-IL-23 and anti-IL-17A directly or indirectly reduces the production of IL-17A^28^. Hence, it is suggested that anti-TNF therapy can inhibit IL-17A production.

IFN-γ is another cytokine involved in the pathogenesis of psoriasis, and it plays an important role in the inflammatory process of the disease, as it seems to be associated with the perpetuation of the inflammatory process by having a synergistic effect with IL-17A in the activation of keratinocytes^41^. Studies have shown that increased serum levels of IFN-γ are directly associated with active disease^42^. The use of biological therapies has also been shown to be effective in reducing IFN-γ levels, as they reduce the activity of TNF-α-producing Th1 cells, and therefore, in reducing IFN-γ levels^43^. In our study, stimulation with anti-CD3 and anti-CD28 was able to significantly increase IFN-γ levels only in day 0, in which the assays were performed just before the pulse therapy with anti-TNF. On the contrary, stimulation with anti-CD3 and anti-CD28 7 days after anti-TNF therapy was not able to significantly increase IFN-γ levels. Investigating the number of IFN-γ^+^CD4^+^ T lymphocytes, we observed that anti-CD3 and anti-CD28 stimulation induced a significant increase in the number of IFN-γ-producing cells only in day 0, suggesting that IFN-γ-producing cells are more resistant to the TNF blockage.

Like IL-17 and IFN-γ, TNF-α is a crucial cytokine in the inflammatory process of psoriasis, and it is produced by cells of both the innate and adaptive immune systems. Circulating levels of TNF-α, IFN-γ, and IL-17A are directly correlated with the severity of psoriasis^44^. Biological therapy has been shown to reduce TNF-α levels in most studies. Anti-TNF-α medications block the free soluble fraction and membrane fraction of this cytokine^45^. In this study, anti-CD3 and anti-CD28 stimulation significantly increased TNF-α production only in day 0, on which the assays were performed just before anti-TNF pulse therapy. These data are consistent with the literature that has demonstrated that immunosuppressive medications such as methotrexate and anti-TNF can reduce the levels of this interleukin during treatment^46^.

In addition to the participation of proinflammatory cytokines involved in psoriasis, the role of anti-inflammatory cytokines has also been investigated. IL-10 plays a critical role in protecting against tissue damage in acute inflammatory processes^47^, thus suppressing the expression of inflammatory cytokines produced by effector cells^48^. Studies indicate that psoriasis patients are deficient in IL-10^49^. Some authors suggest that there is a mild deficiency in the expression of IL-10 messenger RNA in psoriasis in comparison with other inflammatory skin diseases^50^. In this study, under anti-CD3 and anti-CD28 stimulation, IL-10 was also found to increase significantly just before anti-TNF pulse therapy, hence suggesting that the regulatory immune mechanism was able to be elicited in these patients, even though it was not sufficient to block the proinflammatory cytokines that were produced. Therefore, these data suggest that the use of anti-TNF interferes with the immune response, negatively modulating the production of IL-17, IFN-γ, TNF-α, and IL-10 after *in vitro* stimulation with anti-CD3 and anti-CD28. This modulatory property was also demonstrated in an *in vitro* model, where adding anti-TNF-α antibodies to PBMC cultures downregulated the production of IL-17, IFN-γ, TNF-α, and IL-10^51^.

Interestingly, the different TNF inhibitors appear to have distinct mechanisms of action. Anti-TNF has been shown to act more quickly on Th17 than on Th1 populations, and the inhibition of the Th1 population seems to have a stronger correlation with clinical improvement^28^.

The analysis of regulatory T cells (CD25^hi^FoxP3^+^LAP^+^) after *in vitro* activation of CD4^+^ T cells with anti-CD3 and anti-CD28 demonstrated that anti-TNF therapy modulates the number of cells of the Treg phenotype, indicating that the number of Treg cells is reduced during the pulse therapy. These results suggest that the ability to expand Treg cells *in vitro* is modulated by anti-TNF pulse therapy; however, it appears to be restored by the end of the therapeutic treatment. Although some studies have shown that there is a decrease in the number and function of Tregs in psoriasis^21^, other studies have shown an increase in the number of CD4^+^CD25^+^FoxP3^+^ regulatory T cells in peripheral blood *ex vivo*^52,53^. This is the first study evaluating LAP+ Treg cells in psoriasis. The evaluation of the time course of treatment with anti-TNF showed inhibition of the activation of CD4^+^ T cells. Moreover, that the down modulation of IFN-γ -producing cells are highly dependent of anti-TNF therapy, while IL-17 –producing cells remain stable down regulated at the 2 time point of treatment analyzed. Due T regulatory-derived TGF-β acts in immunoregulatory mechanisms and Th17 differentiation^25^, the possible link between LAP expression in T cells and suppressive versus IL-17 induction activity needs to be further characterized in psoriasis.

In this study, we also evaluated patients on methotrexate and anti-TNF therapy. It was observed that, regardless of the treatment approach, TNF-α levels were significantly lower in relation to the control group after *in vitro* stimulation. The role of immunobiological agents in the reduction of TNF-α by is well documented^28,35,54^. Methotrexate is an immunosuppressive folic acid antagonist that is able to inhibit the activation of lymphocytes and macrophages, thus modulating cytokines and inhibiting neutrophil chemotaxis^55^. Studies have also shown that methotrexate is able to block TNF-α production by T cells and macrophages^56^. In this study, low levels of TNF-α were observed even in untreated patients. It is important to emphasize that this group comprised patients that had a stable chronic psoriasis profile despite not undergoing therapy for at least 30 days. In addition, it must be noted that some of these patients had previously underwent methotrexate treatment, which was unsuccessful. Furthermore, it is important to note that all patients who received TNF blockers were previously treated with methotrexate and had poor therapeutic response. These results suggest that both TNF blockers and methotrexate are capable of modulating TNF-α synthesis. Furthermore, patients with stable psoriatic disease retain the ability to modulate TNF-α production. IL-6 is also a proinflammatory cytokine, and it plays a key role in the differentiation of Th17 cells and in the activation of myeloid DCs^57^. Studies have shown the correlation between the levels of this cytokine and disease activity, as well as its regulation in immunosuppressive therapies^58^. Our study showed that treatments both with methotrexate and immunobiologicals significantly reduced the stimulated production of IL-6.

Interestingly, in our results, patients on methotrexate or anti-TNF therapy produced less IL-10 than the control group. These data suggest that these therapeutic strategies simultaneously affect the production of pro-inflammatory cytokines and anti-inflammatory cytokines such as IL-10. Thus, our results suggest that the beneficial effects of the treatment are not associated with a higher production of IL-10 or development of Treg cells, but rather with the direct effects of both methotrexate and immunobiologicals on the blockage of other inflammatory cytokines, such as TNF-α, IL-17, and IFN-γ.

In this study, the expression of cell activation markers and intracellular cytokines in CD4^+^ T cells in untreated and treated patients was compared with the control group. It was observed that patients on methotrexate or anti-TNF therapy had a lower number of activated CD4^+^ T cells after anti-CD3 and anti-CD28 stimulation than the control group. Evaluation of intracellular cytokines in these cells showed no differences between IFN-γ^+^, IL-17^+^, or IL-17^+^IFN-γ^+^ double-positive populations. Our results indicate that both the treatment with methotrexate and with TNF inhibitors negatively modulates CD4^+^ T cell activation, but does not interfere with their ability to produce IL-17 or IFN-γ. The increase in the number of cells with a Treg phenotype in untreated patients may indicate a functional failure of these cells, at least in patients with stable psoriatic disease.

## Conclusions

Treatment with IBs and methotrexate modulates the activation of CD4^+^ T cells. Anti-TNF appears to have a modulatory effect on the activation and production of cytokines by Th1, Th17, and Treg lymphocytes in a distinct manner. Down modulation of IFN-γ-producing cells are highly dependent on anti-TNF therapy, while IL-17-producing cells remain stable down regulated at the 2 time point of treatment analyzed.
